# Editorial: Artificial intelligence in microbial and microscopic analysis

**DOI:** 10.3389/fmicb.2026.1909573

**Published:** 2026-07-20

**Authors:** Jinghua Zhang, Jia Guo, Qing Liu, Fan Liu

**Affiliations:** 1 College of Computer Science and Software Engineering, Hohai University, Nanjing, China; 2 School of Electronic Engineering and Computer Science, Queen Mary University of London, London, United Kingdom; 3 Department of Physics and Engineering, UiT The Arctic University of Norway, Tromsø, Norway

**Keywords:** artificial intelligence, deep learning, machine learning, microbiology, microscopic image analysis

Artificial intelligence (AI) is transforming the way researchers observe, quantify, and interpret microbial and microscopic systems. Microscopy, sequencing, physiological signals, clinical records, and multi-source biomedical data now provide increasingly rich descriptions of biological and clinical processes. However, their complexity also challenges conventional manual or rule-based analysis. AI-driven methods offer new opportunities to extract discriminative features, integrate heterogeneous information, improve diagnostic and monitoring workflows, and generate reproducible evidence across microbiology, microscopic imaging, bioinformatics, and clinical decision support.

The Research Topic “*Artificial intelligence in microbial and microscopic analysis*” was launched to collect methodological advances and practical applications that connect AI with microbial, microscopic, and biomedical analysis. The 15 articles in this Research Topic demonstrate the breadth of this interdisciplinary field, spanning environmental microorganism recognition, antimicrobial resistance surveillance, microbiome-based disease prediction, medical microscopic image analysis, multimodal single-cell analysis, physiological signal interpretation, precision measurement, and domain-specific AI systems. Together, these contributions show how AI can support both biological discovery and clinically oriented decision-making.

A significant portion of the contributions focused on environmental microorganism image analysis, where microscopic images provide direct morphological evidence for ecological monitoring and public health assessment. Guo et al. constructed a reproducible benchmark for environmental microorganism object detection on EMDS-7, evaluating 25 representative detectors under a unified protocol and showing that precise localization and false-positive suppression remain central challenges in cluttered microscopic scenes. Li et al. proposed WPF-Mamba, a wavelet-based progressive multispectral fusion framework for fine-grained microorganism detection, demonstrating the value of spectral alignment and multi-scale feature refinement for small and visually similar microbial objects. Zhou J. et al. established EMDS-7-FSCIL, the first benchmark for few-shot class-incremental learning in environmental microorganism recognition, revealing that methods that are successful on generic visual benchmarks do not directly transfer to dynamic microorganism recognition scenarios. Xu et al. further addressed this problem by proposing a dual-graph knowledge distillation framework that preserves instance-and class-level relational knowledge while adapting to new microorganism categories with limited labeled samples. Collectively, these studies provide datasets, benchmarks, and algorithmic directions for scalable, reproducible, and adaptive microorganism recognition.

Several articles extended AI from microscopic observation to infectious disease characterization, antimicrobial decision-making, and microbiome-related risk prediction. Qu et al. reviewed how AI can support intensive care infectious disease decisions, emphasizing actionable outputs such as calibrated probabilities, safe deferral under uncertainty, and endpoints linked to antimicrobial stewardship. Bai et al. proposed TRACE, a triple-based reconstruction framework that extracts organism-specimen-susceptibility evidence from unstructured clinical narratives, helping to recover fragmented antimicrobial resistance signals and characterize rare pathogen-drug phenotypes. He et al. investigated cross-kingdom microbial signatures in colorectal adenomas using shotgun metagenomics and developed a predictive model that integrates bacterial, fungal, archaeal, and viral features for early adenoma detection. Peng et al. designed a 1DCNN-BiLSTM-Transformer model for hypertension risk prediction based on arterial pressure waveforms, linking physiological signal modeling with microbiome-related pathophysiological interpretation. Zhou Y. et al. developed an XGBoost-based model to predict stroke risk in Chinese hypertensive patients, showing improved discrimination over traditional risk scales. Liu et al. integrated multi-source clinical, cohort, and animal data to reveal the predictive value of inflammatory markers in osteoporosis. Together, these contributions illustrate that AI can connect microbial and biomedical evidence from imaging, omics, text, physiological signals, and clinical variables, thereby supporting more precise risk stratification and decision-making.

Another set of studies advanced AI methods for medical microscopic analysis, multimodal biological data integration, and broader biomedical intelligence. Li proposed an uncertainty-driven global-local Mamba with UNet for gastric cancer histopathological image classification, combining uncertainty-guided feature selection, long-range dependency modeling, local texture extraction, and multi-scale fusion to support computer-aided pathology. Xingzuo et al. introduced a structure-guided soft deep clustering framework for integrating scRNA-seq and scATAC-seq data, enabling probabilistic cell assignments and improving the characterization of cellular heterogeneity. Ding et al. proposed MCEEGNet, a multi-cue EEG network for quantitatively assessing depression using emotional stimulus-induced EEG signals. This extends AI-based diagnosis from binary classification toward severity estimation. Wang et al. developed a Malus's law-enhanced Michelson interferometer with particle swarm optimization-based calibration and Gaussian process regression-based error compensation, providing an AI-assisted measurement paradigm for medical micro-displacement monitoring. Peng and Pan proposed HCE-RAG, a hierarchical context enhancement method for long-tail entity retrieval-augmented generation, addressing semantic drift in domain-specific question answering and highlighting the importance of robust knowledge retrieval for specialized biomedical and scientific contexts. These studies broaden the scope of the Research Topic by demonstrating that AI-enabled microscopic and biomedical analysis depends on more than just recognition models; it also depends on uncertainty modeling, multimodal fusion, intelligent measurement, and reliable knowledge retrieval.

Across the Research Topic, several common themes emerge. First, data scarcity, long-tail distributions, and annotation cost are recurring bottlenecks, particularly in microorganism recognition, rare antimicrobial resistance phenotypes, and clinical prediction. These issues are closely aligned with the broader motivation of few-shot class-incremental learning, which aims to learn new classes from sparsely labeled samples while preserving previously acquired knowledge. This paradigm is particularly relevant to biomedical and microscopic scenarios, where new categories may continuously emerge; however, large-scale expert annotations are difficult to obtain. Second, general-purpose AI models often require domain-specific adaptation because microscopic and biomedical data contain subtle morphology, noisy acquisition conditions, heterogeneous modalities, and clinically meaningful but low-frequency patterns. Third, reproducibility and benchmarking are essential for progress, as demonstrated by studies that provide unified protocols, systematic comparisons, and externally validated prediction models. Finally, interpretability, calibration, and uncertainty estimation are becoming increasingly important, especially when AI outputs are intended to support laboratory, ecological, or clinical decisions.

Looking forward, AI in microbial and microscopic analysis will likely move toward more integrative and trustworthy workflows. Foundation models, self-supervised learning, few-shot learning, class-incremental learning, multimodal fusion, and retrieval-augmented systems may reduce dependence on large, annotated datasets while improving adaptability to new organisms, diseases, instruments, and clinical environments. In addition, future multi-source biomedical AI systems should move beyond simple feature concatenation and incorporate domain knowledge into model architecture, feature interaction, and spatial or biological consistency constraints. At the same time, future studies should place stronger emphasis on external validation, open benchmarks, transparent reporting, and collaboration between computational researchers and domain experts. These efforts will be essential for translating AI methods from retrospective datasets into robust tools for microbiology, microscopy, pathology, and precision medicine.

In conclusion, the articles collected in this Research Topic demonstrate the expanding role of AI in microbial and microscopic analysis. By bringing together advances in environmental microorganism recognition, infectious disease intelligence, microbiome-based prediction, medical image analysis, multimodal biological data mining, and domain-specific AI systems, this Research Topic provides a broad view of how computational intelligence can enhance biological discovery and biomedical decision-making. We hope that this Research Topic will stimulate further interdisciplinary research and promote the development of reliable, interpretable, and deployable AI tools for the microscopic life sciences.

